# Are you coping how I'm coping? An exploratory factor analysis of the Brief-COPE among caregivers of children with and without learning disabilities during COVID-19 restrictions in the UK

**DOI:** 10.1080/20473869.2024.2359134

**Published:** 2024-06-04

**Authors:** Freyja Steindorsdottir, Karen Goodall, Hope Christie, Doug McConachie, Jo Van Herwegen, Carrie Ballantyne, Caroline Richards, Hayley Crawford, Laura Outhwaite, Thomas Gallagher-Mitchell, Joanna Moss, Grace Khawam, Karri Gillespie-Smith

**Affiliations:** a Department of Health and Clinical Psychology, School of Health in Social Science, University of Edinburgh, Edinburgh, UK; b Institute of Education, University College London, London, UK; c Division of Psychology, School of Education and Social Science, University of West of Scotland, Ayr, UK; d School of Psychology, University of Birmingham, Birmingham, UK; e Division of Health Sciences, Warwick Medical School, Warwick University, Coventry, UK; f Department of Psychology, Liverpool Hope University, Liverpool, UK; g School of Psychology, University of Surrey, Guildford, UK

**Keywords:** COVID-19, learning disabilities, caregivers, coping, Brief-COPE, mental health

## Abstract

**Objectives:**

The current study aimed to answer the following questions: (1) What factor structures emerge to responses to the brief coping orientation to problems experienced inventory in a post-pandemic population of caregivers of children under 18 in the UK? (2) What differences, if any, are there in coping between parents of children with and without Learning Disabilities during COVID-19 restrictions?

**Methods:**

An exploratory factor analysis was conducted across a sample of parents of children with and without learning disabilities. Following this, differences between responses on each factor were assessed with Mann Whitney *U* tests.

**Results:**

The analysis recovered six factors across the whole sample: External support-seeking, emotion-focused disengagement, positive cognitive reframing, substance use, religion, and problem-focused disengagement. These structures are largely in line with previous findings. Significant differences were found between groups on the emotion-focused disengagement factor, with parents of children with learning disabilities scoring significantly higher on measures of self-blame, denial, and one venting item.

**Conclusions:**

These findings suggest some consistency in recovered factor structures of the Brief-COPE. Further work exploring the role of emotion-focused disengagement in caregivers of children with Learning Disabilities could provide further insight into what support may need to be made available in this group.

## Introduction

Emerging literature on the psychological impact of the COVID-19 pandemic suggests increased rates of distress and decreased well-being across the population, with high rates of anxiety, loneliness, and psychiatric disorders linked to social isolation due to lockdown measures reported in the early stages of the pandemic (Banerjee and Rai 2020; Knox et al. 2022; Li and Wang 2020; Office for National Statistics 2020). Reduced social contact, health-related anxiety, and time spent in quarantine have been linked previously to decreased psychological well-being (Bai et al. 2018; Hawryluck et al. 2004) and time spent in lockdown was positively correlated with rates of stress, anxiety, and depression (Ozamiz-Etxebarria et al. 2020).

Families with children faced substantial threats to their well-being as they navigated complex lifestyle changes due to school and activity closures and remote working requirements (Calvano et al. 2021; Knox et al. 2022). School closures and restrictions forced caregivers to restructure routines, provide adequate technology for home learning, and navigate how to communicate with their children about the pandemic (Thorell et al. 2021). Parents who had children with learning disabilities faced additional barriers to everyday life due to difficulty accessing adequate support before and during the pandemic (Jeste et al. 2020; Stefanidis, King-Sears, and Kyriakidou 2021) as well as managing complex family dynamics (e.g. sibling conflicts, Toseeb et al. 2020). Caregivers also reported increased levels of behaviours that challenge during COVID-19 restrictions in children who have Special Educational Needs (Nisha and Bakul 2022) which can indicate the child’s increased mental distress (Courtenay and Perera 2020).

These factors may lead to poor mental health in caregivers of children with learning disabilities with most reporting higher rates of depression (52.3%), anxiety (52.3%), and stress (41.1%), compared to the general population (33.7, 31.6, and 29.6%, respectively) (Lim et al. 2021). In addition, several countries (e.g. UK and Italy) have reported significantly higher levels of poor mental health in caregivers of children with Learning Disabilities compared to caregivers of neurotypical children (Gillespie-Smith et al. 2021; Zampini et al. 2024). Additionally, studies have shown that coping strategies adopted by caregivers during COVID-19 contribute to poorer mental health outcomes. For example, Gillespie-Smith et al. (2021) reported that during initial COVID-19 lockdown phases, higher levels of distress in caregivers were predicted by both child behaviours that challenge and caregivers engaging in behavioural disengagement coping. Additionally, Moraes Silva Gomes et al. (2023) found that caregivers of people who have Learning Disabilities and used more emotion focused coping strategies during COVID-19 lockdown had worse mental health outcomes. Taken together these studies show that caregiver coping is an important factor in predicting poor mental health outcomes during the COVID-19 pandemic.

Since Coping strategies predict mental health outcomes in caregivers, it is important to ensure that coping measures are adequately capturing these strategies. One of the most commonly utilised measures of coping in the literature is the Brief Coping Orientation to Problems Experienced inventory (Brief-COPE), shortened from the full-length COPE inventory (Carver 1997), which was developed based on Lazarus and Folkman’s (1984) transactional theory of stress and coping and Carver and Scheier (1981) model of behavioural self-regulation. The Brief-COPE is a 28 item questionnaire with 14 proposed first-order subscales each formed of two items rated on a 4-point Likert scale (1 = I haven’t been doing this at all to 4 = I've been doing this a lot). An overview of the 14 subscales of the Brief-COPE is outlined in Table 1. The Brief-COPE has been utilised in research on coping strategies in families of children with and without Learning Disabilities, finding associations between coping strategies and maternal mental health (Ganjiwale et al. 2016; Hastings et al. 2005; Panicker and Ramesh 2019). Specifically, problem-focused coping strategies are associated with higher positive affect (Adams et al. 2018), and active avoidance and religious/denial coping are associated with mental health problems (Agha 2021).

**Table 1.** Coping strategy subscales from the Brief-COPE (Carver 1997).

Coping strategy	Behaviour	Item
Active coping	Taking steps to eliminate the stressor or its effects	2. I've been concentrating my efforts on doing something about the situation I'm in. 7. I've been taking action to try to make the situation better.
Instrumental support	Seeking out information, help, advice	10. I’ve been getting help and advice from other people. 23. I’ve been trying to get advice or help from other people about what to do.
Positive reframing	Reframing a stressful event in a positive way	12. I've been trying to see it in a different light, to make it seem more positive. 17. I've been looking for something good in what is happening.
Planning	Thinking about how to handle a stressor	14. I've been trying to come up with a strategy about what to do. 25. I've been thinking hard about what steps to take.
Emotional support	Seeking understanding or sympathy from others	5. I've been getting emotional support from others. 15. I've been getting comfort and understanding from someone.
Venting	Discussing feelings about the stressful situation	9. I've been saying things to let my unpleasant feelings escape. 21. I've been expressing my negative feelings.
Humour	Making jokes about the situation	18. I've been making jokes about it. 28. I've been making fun of the situation.
Acceptance	Acknowledging reality of the situation	20. I've been accepting the reality of the fact that it has happened. 24. I've been learning to live with it.
Religion	Turning to faith	22. I've been trying to find comfort in my religion or spiritual beliefs. 27. I've been praying or meditating.
Self-blame	Attributing negative responsibility to oneself for the situation	13. I’ve been criticizing myself. 26. I’ve been blaming myself for things that happened.
Self-distraction	Focusing on alternative activities	1. I've been turning to work or other activities to take my mind off things. 19. I've been doing something to think about it less, such as going to movies, watching TV, reading, daydreaming, sleeping, or shopping.
Denial	Refusing to acknowledge or believe the situation	3. I've been saying to myself ‘this isn’t real’. 8. I've been refusing to believe that it has happened.
Substance use	Taking alcohol or drugs to deal with the stressor	4. I've been using alcohol or other drugs to make myself feel better. 11. I've been using alcohol or other drugs to help me get through it.
Behavioural disengagement	Helplessness or inaction	6. I've been giving up trying to deal with it. 16. I've been giving up the attempt to cope.

Research utilising the Brief-COPE faces limitations regarding generalisability across studies due to the inconsistency in higher-order factors, that is, groupings of inventory subscales into factors, such as problem-focused or emotion-focused coping (Kannis-Dymand et al. 2020; Krägeloh 2011; Monzani et al. 2015). Carver (2018) recommends conducting sample-specific data reduction to explore factor structures. However, a systematic review (Solberg, Gridley, and Peters 2021) found studies have identified structures ranging from 2 to 14 factors (mean 6.15). In addition, there are varying approaches to conceptualising the structures (engagement *vs.* disengagement, approach *vs.* avoidance) (Solberg Nes and Segerstrom 2006). However, there has been relative consistency in extracting three factors to some degree: (1) active, positive, or engagement coping, (2) support-seeking, and (3) avoidance, denial, and disengagement (Muniandy et al. 2021). Table 2, adapted from Solberg, Gridley, and Peters (2021), outlines factor analyses that have been conducted on the Brief-COPE in relevant populations.

**Table 2.** Factor analyses of the Brief-COPE in caregivers or COVID-19 populations.

Citation	Country	N and Sample	Analysis	Identified factors
Abdul Rahman, Bani Issa, and Naing (2021)	UAE	423 nurses in Brunei	CFA/EFA	Two factor structure (unnamed)
Baumstarck et al. (2017)	France	398 cancer patients and their caregivers	PCA/CFA	Social support Problem solving Avoidance Positive thinking
Benson (2010)	USA	113 mothers of children with autism	PCA	Problem focused Avoidant Social supported coping Emotion focused coping
Hastings et al. (2005)	UK	135 caregivers of pre-school age children with autism	PCA	Active avoidance coping Problem-focused coping Positive coping Religious/denial coping
Kershaw et al. (2004)	USA	378 patients with breast cancer and their caregivers	EFA	Active coping Avoidant coping
Kimemia, Asner-Self, and Daire (2011)	Kenya	134 Kenyan caregivers	PCA	Emotional and instrumental support Planning, active coping and acceptance Self-blame and behavioural disengagement Religion, positive reframing, and humour Denial and venting
Jathanna, Latha, and Bhandary (2010)	India	125 Dementia caregivers	PCA	Acceptance Humour Religion Behavioural disengagement Substance use Self-blame
Muniandy et al. (2021)	Australia	255 autistic adults, 165 non-autistic adults	EFA	Engagement coping Support-seeking coping Disengagement coping Substance-use coping Humour coping Religious coping
Nunes et al. (2021)	Portugal	153 community parents, 116 at-risk parents	CFA	Original 14 subscales in both samples
Tang, Chan, and Choi (2021)	Hong Kong	217 caregivers of children with chronic illnesses	EFA	Active coping Distraction Dysfunctional coping
Zelikovsky, Schast, and Jean-Francois (2007)	USA	144 parents of children with end-stage renal disease	PCA	Adaptive coping Avoidant coping

*Note:* Adapted from Solberg, Gridley, and Peters (2021).

Only one study so far has explored the dimensionality of the Brief-COPE following the onset of the COVID-19 pandemic, finding support for the original 14-subscales but no higher-order factors in an adult sample (Hanfstingl et al. 2021). How the pandemic has influenced coping mechanisms utilised by caregivers of both children who are neurotypical and children who have Learning Disabilities is of concern as we move away from nationwide restrictions (Solberg, Gridley, and Peters 2021). Further exploration into how factor structures emerge following the onset of the pandemic, especially in vulnerable populations, enables more robust research to be conducted to develop appropriate support and interventions; or alternatively to develop more appropriate and valid measures of coping in a post-pandemic world.

The current study aimed to explore the following:

1. What factor structures emerge to responses to the brief-COPE in a post-pandemic population of caregivers of children under 18 in the UK?
2. What differences, if any, are there in coping between parents of children with and without Learning Disabilities during COVID-19 restrictions?

## Methods

### Project road to recovery

This study was conducted in collaboration with the Road to Recovery project, an ongoing research project exploring the impact of COVID-19 on caregivers of children with and without Learning Disabilities. The project, funded by the Economic and Social Research Council, aimed to capture experiences of children with Learning Disabilities and their caregivers throughout various stages of the pandemic to inform policy and government decision-making, along with aiding in the development of appropriate support (Road to Recovery, 2021).

### Participants and procedure

Utilising data from the Road to Recovery survey (collected between August 2021 to January 2022), exploratory factor analysis was conducted to determine what factor structure best represents coping strategy use amongst families of children with and without Learning Disabilities in the UK (*N* = 175). Participants were eligible to participate if they (1) were a primary caregiver to a child between the ages of 5–18 and (2) lived in the UK. Table 3 outlines the demographic characteristics of the sample which shows that the majority of the sample had a child who also had a co-occurring neurodevelopmental disorder. It was made clear to participants during recruitment that Learning Disability was being used in the UK sense (as a synonym of Intellectual Disability and is characterized by significant limitations in intellectual functioning and adaptive behaviour). For more information about the participants with Learning Disabilities as well as any co-occurring neurodevelopmental conditions please see Supplementary Table I.

**Table 3.** Demographics of the parent groups of children who are neurotypical (NT) and children who have learning disabilities (LD).

	NT group		LD group	
	*n*	*%*	*n*	*%*
Total	78	100	97	100
Child characteristics				
Age, mean in years (*SD*)		11.2 (4.7)	11.9 (4.2)	
Female	34	43.6	31	32.0
Male	43	55.1	65	67.0
Non-binary	1	1.3	1	1.0
Child living situation				
At home with family	75	96.2	92	94.8
At home with carers	2	2.6	4	4.1
Other	1	1.3	1	1.0
Child education				
Hours per week, mean (*SD*)		29.8 (9.3)	26.5 (9.5)	
Mainstream	69	88.5	41	42.3
SEN (non-residential)	6	7.7	42	43.3
SEN (residential)	–	–	3	3.1
Combination	1	1.3	4	4.1
Other	2	2.6	7	7.2
Child education level				
Pre-school	1	1.3	5	5.2
Primary school	43	55.1	50	51.5
Secondary schools	31	39.7	30	30.9
University/College	3	3.8	8	8.2
Not in education	–	–	4	4.1
Caregiver relationship				
Mother	62	79.5	85	87.6
Father	15	19.2	11	11.3
Other	1	1.3	1	1.0
Caregiver work				
Working full-time	34	43.6	25	25.8
Working part-time	27	34.6	34	35.0
Unemployed	–	–	2	2.1
Homemaker	11	14.1	22	22.7
Volunteer work	–	–	2	2.1
Student	1	1.3	3	3.1
Self-employed	3	3.9	2	2.1
Other	2	2.6	2	7.2
Caregiver education				
Postgraduate	22	28.2	21	21.6
Undergraduate	31	39.7	41	42.3
Vocational training	10	12.8	18	18.6
Higher/diploma	12	15.4	12	12.4
Other	2	2.6	3	3.1
None/missing	1	1.3	2	2.1
Caregiver marital status				
Single	6	7.7	13	13.4
Married or in a civil partnership	60	76.9	67	69.1
Living with partner	11	14.1	12	12.4
Divorced/separated	1	1.3	4	4.1

*SD*: standard deviations in parenthesis.

While the specific restrictions varied between the UK nations of Scotland, England, Wales, and Northern Ireland, the main lockdown restrictions were similar throughout the UK, including rules, such as reductions in gatherings, movement, and limited services (Ferguson, Brown, and Barber 2021). Schools were open during the period of data collection however legal restrictions still remained on gatherings, travel, and social isolation were still enforced throughout 2021 with all restrictions finally lifting in January 2022 for Scotland (BBC News 2022a); February 2022 for England and Northern Ireland (BBC News 2022b), and Wales in May 2022 (Culbertson 2022). Following ethical approval from the Department of Clinical Psychology at the University of Edinburgh (Ethics070) as part of the Road to Recovery project, an online survey was developed measuring demographic data, diagnostic information, and questions regarding coping (Brief-COPE, Carver 1997), challenging behaviours (Developmental Behavioural Checklist-Brief, DBCP24, Taffe et al. 2007), psychological distress (Depression and Anxiety Stress Scale, DASS21, Lovibond and Lovibond 1995), attachment patterns in relationships (The Relationship Structures Questionnaire, ECR-RS, Fraley et al. 2011) and aspects of support throughout restrictions. This study extracted demographics and Brief-COPE responses only from the original survey. The survey took 30–45 min to complete. Participants received £20 in Amazon vouchers upon completion.

### Measures

#### Demographics

Participants answered questions regarding their relationship to the child, the child’s diagnostic status (Yes-they have a diagnosis/No), date of birth, and gender, as well as their child’s and their own educational, marital, and work status.

#### Brief-COPE

Participants were asked to complete the situational version of the previously detailed Brief-COPE (Carver 1997); consisting of 28-items rated on a 4-point Likert scale (1 = I haven’t been doing this at all to 4 = I've been doing this a lot) that form 14 subscales. Carver (1997) reports moderate Cronbach’s alpha reliabilities for the 14 subscales (0.50–0.90) with all except Venting, Denial, and Acceptance >0.60, above a general threshold for acceptability but slightly under the ideal of 0.70–90 (Taber 2018; Ursachi, Horodnic, and Zait 2015). To better capture potential differences in structures post-pandemic, this analysis utilised the 28 items instead of the 14 subscales.

### Statistical analyses

#### EFA

A factor analysis was conducted to explore what factor structures emerge from responses to the Brief-COPE in the current full sample. Given previously discussed inconsistencies regarding the factor structure of the Brief-COPE, and the assumption that the items on the Brief-COPE measure latent variables, a common factor model (Exploratory Factor Analysis, EFA) was considered more appropriate than Confirmatory Factor Analysis (CFA) or Principal Component Analysis (PCA) (Watkins 2021; Widaman 2018). As the data was comprised of ordinal variables, both EFAs were conducted using principal axis factoring (PAF) extraction (Watkins 2018). An oblique (oblimin) rotation was chosen as due to the theoretical underpinnings of the scale it was expected that factors would correlate (Carver 1997; Meehl 1990, Watkins 2018).

Guidelines for recommended sample sizes in exploratory factor analyses vary. Factor recovery improves with increasing sample sizes and communalities, yet there is little consensus regarding absolute minimum sample size, though few guidelines describe samples under *N* = 100 (de Winter, Dodou, and Wieringa 2009; Watkins 2021). However, given their exploratory nature, de Winter, Dodou, and Wieringa (2009) suggest that conducting EFAs is better than not conducting them at all, given careful interpretation of findings. Missing cases were excluded pairwise (see table) to prevent loss of power due to the smaller sample size.

Factorability was assessed using multiple methods: firstly, correlation matrices to confirm some moderate correlations (0.3–0.5) (Tabachnick, Fidell, and Ullman 2019). In addition, the Kaiser-Meyer-Olkin (KMO) test of sampling adequacy was used, which assesses whether a sample size is sufficient for factor analysis, with a value of under 0.5 indicating a sample size is too small and an ideal value of over 0.7 (Kaiser 1974; Watkins 2021). Finally, Bartlett’s test of sphericity was conducted which tests the null hypothesis that the correlation matrix is an identity matrix with a significant alpha level of 0.05 indicating suitability (Bartlett 1950). Inspection of Scree plots (Cattell 1966), Kaiser’s (1974) criteria of eigenvalues >1.00, and cumulative percentage of extracted variance was utilised to guide how many factors to retain (Costello and Osborne 2005; Kaiser 1974; Watkins 2021). An optimal solution does not include minor factors with little variance explained, nor blur important distinctions between factors (Watkins 2021). Hair et al. (2019) propose that the utility of a factor solution is key: as such, the overriding criteria for choosing factors to retain are interpretability and theoretical relevance. It is agreed that underextraction is more dangerous than overextraction, as underextraction may obscure true factor structure (Watkins 2021). In identifying salient loadings (i.e. when factor loadings are considered meaningful), it is common to set a threshold of 0.30 (Child 2006; Costello and Osborne 2005; Hair et al. 2019). However, our threshold was raised to 0.35 to account for the small sample size in this study.

Complex loadings (items loading saliently on more than one factor) were considered in light of their interpretability and potential to shed light on changes in coping post-pandemic. In addition, Cronbach’s (1951) alpha reliability for each factor should approach 0.70 as is considered acceptable in the literature (Taber 2018). Once an appropriate factor structure was found, factors were descriptively named, with consideration of previous literature to ensure consistency where possible.

#### Between groups comparison

Once a factor structure had been determined, a two-tailed Mann Whitney *U* Test was conducted to determine whether there was a significant difference in responses to each factor between caregivers of children with and without Learning Disabilities.

## Results

### EFA

First, data was checked to ensure there was enough covariance to justify conducting an EFA. Pearson’s correlation matrices indicated several moderate correlations of ≥0.30 between items and no strong correlations ≥.90. Sampling adequacy was confirmed with an acceptable KMO statistic of .74. Bartlett’s test of sphericity (1950) rejected the null hypothesis that the correlation matrix was an identity matrix *X*^2^ (378) = 1964.57, *p* < .001. Altogether, these measures indicated that the data were appropriate for factor analysis. Kaiser’s (1974) criterion of retaining factors with an eigenvalue >1 indicated that 9 factors could be retained. Inspection of a Scree plot indicated 3–7 factors should be retained (see Supplemental Information Figure 1). Models with 3, 4, 5, 6, and 7 factors were evaluated. In all models, one item (Self-distraction 1) failed to saliently load onto any factors. Of the five models tested, the 5 and 6-factor model was considered the most interpretable with reasonable parameter estimates and no cross-loadings. Self-distraction was excluded and the 5 and 6 factor models re-run. Following this, the 6-factor model was considered most interpretable and chosen as the final model. Sampling adequacy was confirmed with an acceptable KMO statistic of .74. Bartlett’s test of sphericity (1950) rejected the null hypothesis that the correlation matrix was an identity matrix *X*^2^ (351) = 1922.27, *p* < .001. Only the final model is presented here; for additional models please see Supplemental Information Tables ii–vii.

#### Final model

Simpler structure, interpretability of factor structures, and lack of complex loadings led to the 6-factor model with Self-Distraction 1 item excluded being considered most appropriate. The final model is reported in Table 4. Cronbach’s (1951) alpha indicated good reliability of factors with all exceeding .70. Table 5 outlines interfactor correlations for the final model.

**Table 4.** Final factor structures of the Brief-COPE.

Factors	1	2	3	4	5	6
Variance explained (%)						
Cronbach’s alpha						
Emotional support 1	**.844**	−.012	−.021	.055	.150	.085
Use of instrumental support 1	**.773**	.090	−.069	.061	−.124	−.122
Emotional support 2	**.763**	.009	−.024	.123	.103	−.079
Use of instrumental support 2	**.727**	.166	−.069	.118	−.038	−.179
Venting 2	**.568**	.166	.148	−.149	−.010	−.098
Behavioural disengagement 1	−.060	**.778**	.102	−.047	.006	.084
Behavioural disengagement 2	.078	**.766**	−.024	−.043	−.011	.085
Self-blame 2	.156	**.749**	−.028	−.012	−.094	.063
Self-blame 1	.086	**.668**	.167	−.171	.006	.024
Denial 2	−.025	**.620**	−.104	.240	.183	−.170
Denial 1	−.163	**.522**	−.044	.081	.166	−.468
Venting 1	.237	**.463**	.055	.105	.101	.007
Humor 1	−.141	.086	**.721**	.143	.206	−.109
Self-distraction 2	−.003	.216	**.707**	−.082	−.150	.182
Humor 2	.013	.079	**.681**	−.005	.113	.059
Positive reframing 2	.010	−.081	**.524**	.211	−.114	−.309
Acceptance 2	.129	−.201	**.496**	−.050	−.038	−.148
Acceptance 1	.379	−.340	**.424**	−.064	.004	−.039
Religion 2	.012	.009	.062	**.876**	−.059	.086
Religion 1	.167	−.034	−.044	**.868**	−.043	.101
Positive reframing 1	−.050	−.050	.379	.381	−.043	−.211
Substance use 1	.048	−.020	.094	−.047	**.906**	.044
Substance use 2	.076	.023	.009	−.087	**.900**	.044
Planning 1	.125	.016	.019	−.127	−.139	**−.810**
Active coping 2	.020	−.056	.148	−.003	−.076	**−.764**
Active coping 1	.107	−.114	−.096	.036	.176	**−.631**
Planning 2	.292	.009	.067	−.018	−.190	**−.608**

*Note:* Items in bold indicate salient factor loadings >.35. 1—external support seeking, 2—emotion focused disengagement, 3—positive cognitive reframing, 4—religion, 5—substance use, 6—problem focused disengagement. Extraction Method: Principal Component Analysis. Rotation Method: Oblimin with Kaiser Normalization.

**Table 5.** Interfactor correlations for the final model.

Factor	1	2	3	4	5	6
1	1.000					
2	.113	1.000				
3	.195	.018	1.000			
4	.078	.034	.068	1.000		
5	−.008	.209	−.040	.070	1.000	
6	**−.302**	.020	**−**.200	−.237	−.007	1.000

1. External support seeking, 2. Emotion-focused disengagement, 3. Positive cognitive reframing, 4. Religion, 5. Substance use, 6. Problem-focused disengagement. Significant at the *p* < .05 level.

#### Factor naming

*External support-seeking*; comprised of both emotional support items, both instrumental support items and Venting 2, accounted for 18.68% of the variance across the groups. There were no significant differences between groups. *Emotion-focused disengagement*; comprised of both behavioural disengagement items, both self-blame items, both denial items, and Venting 1, accounted for 11.85% of the variance in the sample. *Positive cognitive reframing*; comprised of Humour items, both acceptance items, both positive reframing items, and self-distraction 2, explained 5.88% of the variance across groups. Substance use and Religion items formed their own factors, respectively, in line with previous literature indicating these forms of coping are unique to themselves. The final factor was comprised of Active Coping and Planning items. These items had strong negative loadings onto the factor, indicating the items measure the inverse of the factor, and the factor was named accordingly as *Problem-focused disengagement*; this factor accounted for 3.5% of the variance explained across groups.

### Comparison between groups

Mann-Whitney *U* tests were performed on each of the factors to evaluate whether coping strategies differed by group. The results indicated that the Learning Disability group had significantly higher scores on items relating to ‘Emotion-focused disengagement coping’ than the neurotypical group, *U* = 4121.00, *p* = <.001. There were no significant differences between external support seeking, positive cognitive reframing, substance use, religion, and problem-focused disengagement coping between groups.

## Discussion

The primary aims of this study were to evaluate the factor structure of the Brief-COPE among caregivers of children with and without Learning Disabilities during COVID-19 restrictions in the UK and how this aligns with previous literature, and to explore differences in coping between groups.

### Factor structures

Caregiver coping has been found to directly affect parenting ability and child wellbeing (Adams et al. 2018; Benson 2010) however, research referring to specific coping structures may be referring to different constructs with the same name, or assuming higher-order factors are present in a sample. Before developing effective support for families, it is necessary to have a clear idea of what mechanisms are being utilised, to identify how they affect families. Clearer conceptualisation of coping strategies and how they group together enables more robust research on factors influencing these strategies (such as child behaviours, parenting strategies, and support). The current study recovered 6 factors across the whole sample: *External support-seeking, emotion-focused disengagement, positive cognitive reframing, substance use, religion,* and *problem-focused disengagement.* These structures are largely in line with previous findings. Hanfstingl et al. (2021) found when comparing factor analyses of the Brief-COPE that 7–9 factors may be most adequate for summarizing the scale; despite variation in factor recovery, those most consistently recovered correspond to (1) active, positive, or engagement coping, (2) support-seeking, and (3) avoidance, denial, or disengagement coping (Muniandy et al. 2021).

### Between group differences

Significant differences were found between groups on the emotion-focused disengagement factor, with parents of children with Learning Disabilities scoring significantly higher on items of behavioural disengagement, self-blame, and venting. Previous literature on coping strategies throughout COVID-19 restrictions found that strategies, such as positive reframing, acceptance, and humour were associated with better mental health, whereas venting, self-blame, behavioural disengagement, and self-distraction were associated with mental health difficulties; similar findings have been reported for individuals with disabilities and chronic conditions (Umucu and Lee 2020). Gillespie-Smith et al. (2021) found that parent coping strategies posed an additional risk for distress over and above a child’s diagnosis, and denial and behavioural disengagement were associated with increased levels of distress. This shows that caregiver coping strategies can strongly impact mental health outcomes and could be a potential area to target for intervention and support, improving mental health outcomes.

### Excluded item

Self-distraction 1 (‘I've been turning to work or other activities to take my mind off things’) was excluded as it failed to saliently load onto any factors. Whether this is reflective of COVID-19 restrictions at the time, where access to work and activities may have been limited, or other factors is unclear. The exclusion of self-distraction 1 is not unique to this study: Hastings et al. (2005) excluded the same item in a study of caregivers of autistic children and Muniandy et al. (2021) excluded both self-distraction items in an autistic sample (but not the non-autistic sample). This may suggest that self-distraction items as they are presented do not adequately capture self-distraction techniques and may warrant further exploration.

### Limitations

A key limitation to the study is the small sample size, limiting the generalisability of the results which should be interpreted with caution. A larger sample size may be able to explore patterns between different diagnoses, specific types of child behaviour, or parenting strategies. Secondly, we did not differentiate between type or severity of Learning Disability, and the presence of Learning Disability was self-reported. Understanding the impact of type and severity of Learning Disability on coping strategies would be an avenue for further work. Further, we did not differentiate between age groups of children; understanding how coping strategies may differ between these groups when taking the age group of the child into account would provide better insight into whether coping strategy use differs depending on age group, and whether support provided may need to be adapted in accordance with this. Additionally, while some information regarding demographics was collected, future work in this area might consider collecting more information regarding ethnicity, yearly income, diagnostic or disability status of caregivers, and precise location within the UK, utilising this demographic information in analyses to explore what patterns may emerge. Further, analysis of Brief-COPE responses may be enriched in a mixed-methods design where participants provide examples or explanations for each strategy and have the opportunity to add new strategies. This may shed light on whether items are understood differently or are unclear to participants or whether novel strategies have emerged.

An opportunity to provide examples of strategies not accounted for by the Brief-COPE may assist in capturing novel or alternative coping strategies for further development. It is possible that lockdown environments enabled some opportunities to explore novel approaches to daily life, new skills, and ways to cope through activities, such as walks, baking, and creative pursuits; emerging research on the role of textile arts in dementia care during COVID-19 restrictions, for example, supports this view (Nartker 2022). Further, it is possible that increased time together as a family because of lockdown measures requires increased use of strategies, such as clear communication between family members and boundary setting as a way to cope with increased social demands at home (Gayatri and Irawaty 2022). Extending the Brief-COPE to capture such qualitative changes to coping strategies would allow for a clearer picture of post-pandemic coping.

## Conclusion

Factor structures of the Brief-COPE were largely consistent to previous literature within a sample of caregivers of children with and without Learning Disabilities during COVID-19 restrictions in the UK. Of six recovered factors, external support-seeking explained the most variance. Significant differences were found between groups on the *emotion-focused disengagement* factor with significantly higher scores on measures of self-blame and behavioural disengagement in the Learning Disability group. These findings suggest some consistency in recovered factor structures of the Brief-COPE. Further work exploring the role of emotion-focused disengagement in caregivers of children with Learning Disabilities could provide further insight into what support may need to be made available in this group.

## Supplementary Material

Supplemental Material

## Data Availability

Data currently available on request from the authors however will be openly available after 31 August 2024 on Edinburgh DataShare—https://datashare.ed.ac.uk/.
