# Supplementary material for: Are you coping how I'm coping? An exploratory factor analysis of the Brief-COPE among caregivers of children with and without learning disabilities during COVID-19 restrictions in the UK
Source: Int J Dev Disabil. 2024 Jun 4;72(4):717–28. doi: 10.1080/20473869.2024.2359134 (PMC13202675; doi:10.1080/20473869.2024.2359134)
Supplement: Supplemental Material [file YJDD_A_2359134_SM7444.zip › Table iii_Supplementary.docx]

**Table iii**

*Pattern matrix for 4 factor structure*

| **Factor loading for 4 factor model** | | | | |
| --- | --- | --- | --- | --- |
|  | Factor | | | |
|  | 1 | 2 | 3 | 4 |
| Eigenvalue | 4.981 | 3.173 | 1.542 | 1.330 |
| % Variance explained | 17.79 | 11.33 | 5.51 | 4.75 |
| Use of instrumental support 2 | **.812** | .212 | -.098 | -.033 |
| Use of instrumental support 1 | **.808** | .148 | -.139 | -.110 |
| Emotional support 2 | **.750** | .079 | -.082 | .086 |
| Emotional support 1 | **.683** | .111 | -.123 | .109 |
| Planning 2 | **.588** | -.055 | .202 | -.152 |
| Planning 1 | **.529** | -.093 | .212 | -.121 |
| Venting 2 | **.472** | .205 | .078 | -.011 |
| Active coping 2 | **.419** | -.158 | **.357** | -.069 |
| Active coping 1 | **.411** | -.148 | .119 | .106 |
| Religion 1 | .232 | -.025 | .120 | .027 |
| Behavioural disengagement 1 | -.121 | **.746** | .072 | .004 |
| Behavioural disengagement 2 | .027 | **.733** | -.077 | -.005 |
| Self-blame 2 | .110 | **.716** | -.060 | -.065 |
| Self-blame 1 | .015 | **.612** | .095 | .022 |
| Denial 2 | .129 | **.515** | .016 | .145 |
| Venting 1 | .204 | **.424** | .052 | .083 |
| Denial 1 | .145 | **.363** | .117 | .114 |
| Humor 1 | -.098 | .068 | **.690** | .136 |
| Positive reframing 2 | .170 | -.114 | **.609** | -.086 |
| Self distraction 2 | -.157 | .228 | **.511** | -.106 |
| Humor 2 | -.047 | .105 | **.490** | .037 |
| Positive reframing 1 | .123 | -.088 | **.445** | -.005 |
| Acceptance 2 | .142 | -.160 | **.407** | -.041 |
| Acceptance 1 | .274 | -.234 | .310 | -.008 |
| Religion 2 | .119 | -.010 | .215 | .018 |
| Self distraction 1 | .004 | .127 | .162 | .043 |
| Substance use 1 | -.048 | -.022 | .056 | **.866** |
| Substance use 2 | -.013 | .024 | -.047 | **.851** |
