# Supplementary material for: Are you coping how I'm coping? An exploratory factor analysis of the Brief-COPE among caregivers of children with and without learning disabilities during COVID-19 restrictions in the UK
Source: Int J Dev Disabil. 2024 Jun 4;72(4):717–28. doi: 10.1080/20473869.2024.2359134 (PMC13202675; doi:10.1080/20473869.2024.2359134)
Supplement: Supplemental Material [file YJDD_A_2359134_SM7444.zip › Table ii_Supplementary.docx]

**Table ii**

*Pattern matrix for 3 factor structure*

|  | | | |
| --- | --- | --- | --- |
|  | Factor | | |
|  | 1 | 2 | 3 |
| Eigenvalue | 4.973 | 3.090 | 1.519 |
| Variance explained (%) | 17.76 | 11.04 | 5.42 |
| Use of instrumental support 2 | **.820** | .188 | -.107 |
| Use of instrumental support 1 | **.818** | .086 | -.150 |
| Emotional support 2 | **.741** | .121 | -.079 |
| Emotional support 1 | **.674** | .165 | -.121 |
| Planning 2 | **.598** | -.135 | .193 |
| Planning 1 | **.535** | -.154 | .206 |
| Venting 2 | **.483** | .193 | .066 |
| Active coping 2 | **.420** | -.189 | **.355** |
| Active coping 1 | **.397** | -.087 | .126 |
| Religion 1 | .225 | -.007 | .126 |
| Behavioural disengagement 1 | -.092 | **.717** | .052 |
| Behavioural disengagement 2 | .053 | **.701** | -.094 |
| Self-blame 2 | .138 | **.650** | -.080 |
| Self-blame 1 | .040 | **.600** | .075 |
| Denial 2 | .132 | **.583** | .015 |
| Venting 1 | .210 | **.459** | .048 |
| Denial 1 | .147 | **.417** | .117 |
| Substance use 2 | -.045 | **.392** | -.018 |
| Substance use 1 | -.067 | **.357** | .060 |
| Humor 1 | -.107 | .144 | **.702** |
| Positive reframing 2 | .174 | -.153 | **.606** |
| Humor 2 | -.049 | .127 | **.495** |
| Self distraction 2 | -.131 | .165 | **.485** |
| Positive reframing 1 | .122 | -.086 | **.446** |
| Acceptance 2 | .144 | -.178 | **.403** |
| Acceptance 1 | .273 | -.234 | .308 |
| Religion 2 | .113 | .003 | .221 |
| Self distraction 1 | .009 | .146 | .157 |
