# Supplementary material for: Are you coping how I'm coping? An exploratory factor analysis of the Brief-COPE among caregivers of children with and without learning disabilities during COVID-19 restrictions in the UK
Source: Int J Dev Disabil. 2024 Jun 4;72(4):717–28. doi: 10.1080/20473869.2024.2359134 (PMC13202675; doi:10.1080/20473869.2024.2359134)
Supplement: Supplemental Material [file YJDD_A_2359134_SM7444.zip › Table iv_Supplementary.docx]

**Table iv**

*Pattern matrix for 5 factor structure*

|  | | | | | |
| --- | --- | --- | --- | --- | --- |
|  | Factor | | | | |
|  | 1 | 2 | 3 | 4 | 5 |
| Eigenvalue | 5.005 | 3.187 | 1.564 | 1.344 | 1.261 |
| % variance explained | 17.88 | 11.38 | 5.58 | 4.80 | 4.51 |
| Use of instrumental support 1 | **.808** | .131 | -.127 | -.098 | -.019 |
| Use of instrumental support 2 | **.792** | .199 | -.106 | -.027 | .059 |
| Emotional support 2 | **.739** | .062 | -.088 | .094 | .039 |
| Emotional support 1 | **.699** | .085 | -.097 | .126 | -.065 |
| Planning 2 | **.580** | -.067 | .170 | -.149 | .093 |
| Planning 1 | **.533** | -.106 | .183 | -.117 | .061 |
| Venting 2 | **.524** | .171 | .127 | .012 | -.136 |
| Active coping 2 | **.403** | -.162 | .300 | -.074 | .158 |
| Active coping 1 | **.382** | -.146 | .067 | .099 | .151 |
| Behavioural disengagement 1 | -.109 | **.738** | .108 | .008 | -.035 |
| Behavioural disengagement 2 | .033 | **.727** | -.039 | .000 | -.043 |
| Self-blame 2 | .109 | **.711** | -.028 | -.061 | -.020 |
| Self-blame 1 | .057 | **.591** | .159 | .038 | -.132 |
| Denial 2 | .062 | **.551** | -.046 | .125 | .254 |
| Venting 1 | .189 | **.422** | .046 | .080 | .071 |
| Denial 1 | .106 | **.377** | .070 | .100 | .180 |
| Humor 1 | -.087 | .055 | **.648** | .131 | .137 |
| Self distraction 2 | -.101 | .202 | **.586** | -.094 | -.131 |
| Positive reframing 2 | .154 | -.118 | **.545** | -.095 | .201 |
| Humor 2 | -.005 | .079 | **.508** | .047 | -.034 |
| Acceptance 2 | .177 | -.188 | **.416** | -.028 | -.024 |
| Positive reframing 1 | .065 | -.073 | **.360** | -.025 | .306 |
| Acceptance 1 | .321 | -.274 | .337 | .013 | -.078 |
| Self distraction 1 | .012 | .119 | .171 | .046 | .006 |
| Substance use 1 | -.030 | -.048 | .069 | **.877** | -.022 |
| Substance use 2 | .009 | -.002 | -.022 | **.863** | -.062 |
| Religion 2 | -.090 | .064 | .038 | -.040 | **.770** |
| Religion 1 | .051 | .041 | -.079 | -.023 | **.738** |
