# Supplementary material for: Are you coping how I'm coping? An exploratory factor analysis of the Brief-COPE among caregivers of children with and without learning disabilities during COVID-19 restrictions in the UK
Source: Int J Dev Disabil. 2024 Jun 4;72(4):717–28. doi: 10.1080/20473869.2024.2359134 (PMC13202675; doi:10.1080/20473869.2024.2359134)
Supplement: Supplemental Material [file YJDD_A_2359134_SM7444.zip › Table i_Supplementary.docx]

| **Table i** |  |  |  |  |
| --- | --- | --- | --- | --- |
| *Diagnosis as a percentage of the group with Learning Disability (Learning Disability is being used in the UK sense as a synonym of Intellectual Disability)* | | | | |
|  | n = 97 | |  |  |
| Diagnosis | Number | Percentage |  |  |
| Autism & Learning Disability | 29 | 29.9 |  |  |
| Autism & ADHD & Developmental Delay | 10 | 10.3 |  |  |
| ADHD & Learning Disability | 3 | 3.1 |  |  |
| Down Syndrome | 12 | 12.4 |  |  |
| Williams Syndrome | 12 | 12.4 |  |  |
| Learning Disability | 11 | 11.3 |  |  |
| Global/Developmental Delay | 5 | 5.1 |  |  |
| Williams Syndrome and Autism | 2 | 2.1 |  |  |
| Rare Chromosome Abnormality | 2 | 2.1 |  |  |
| Fragile X Syndrome | 2 | 2.1 |  |  |
| Coffin Siris Syndrome & Autism | 1 | 1.0 |  |  |
| Down Syndrome & Autism | 3 | 3.1 |  |  |
| Rubinstein-Taybi Syndrome & Autism | 1 | 1.0 |  |  |
| Cornelia de Lange Syndrome | 4 | 4.1 |  |  |
