# Supplementary material for: Are you coping how I'm coping? An exploratory factor analysis of the Brief-COPE among caregivers of children with and without learning disabilities during COVID-19 restrictions in the UK
Source: Int J Dev Disabil. 2024 Jun 4;72(4):717–28. doi: 10.1080/20473869.2024.2359134 (PMC13202675; doi:10.1080/20473869.2024.2359134)
Supplement: Supplemental Material [file YJDD_A_2359134_SM7444.zip › Table vii_Suppementary.docx]

**Table vii: Correlations**

|  | Self distraction 1 | Active coping 1 | Denial 1 | Substance use 1 | Emotional support 1 | Behavioural disengagement 1 | Active coping 2 | Denial 2 | Venting 1 | Use of instr. support 1 | Substance use 2 | Positive reframing 1 | Self-blame 1 |
| --- | --- | --- | --- | --- | --- | --- | --- | --- | --- | --- | --- | --- | --- |
| Self distraction 1 | 1 | .192* | 0.061 | 0.092 | .182* | .150* | 0.035 | 0.053 | 0.113 | -0.024 | 0.051 | 0.034 | .156* |
| Active coping 1 | .192* | 1 | .156* | 0.075 | .288** | -0.086 | .442** | 0.134 | 0.033 | .222** | 0.06 | .168* | -0.102 |
| Denial 1 | 0.061 | .156* | 1 | .164* | 0.095 | .303** | .177* | .507** | .252** | .151* | .173* | 0.087 | .215** |
| Substance use 1 | 0.092 | 0.075 | .164* | 1 | 0.096 | 0.12 | -0.048 | .226** | 0.091 | -0.056 | .762** | 0.038 | .216** |
| Emotional support 1 | .182* | .288** | 0.095 | 0.096 | 1 | 0.028 | .184* | .213** | .310** | .615** | 0.113 | 0.072 | 0.095 |
| Behavioural disengagement 1 | .150* | -0.086 | .303** | 0.12 | 0.028 | 1 | -0.063 | .395** | .281** | 0.029 | .238** | -0.039 | .475** |
| Active coping 2 | 0.035 | .442** | .177* | -0.048 | .184* | -0.063 | 1 | 0.053 | 0.073 | .293** | -0.096 | .253** | -0.05 |
| Denial 2 | 0.053 | 0.134 | .507** | .226** | .213** | .395** | 0.053 | 1 | .361** | 0.142 | .180* | 0.048 | .239** |
| Venting 1 | 0.113 | 0.033 | .252** | 0.091 | .310** | .281** | 0.073 | .361** | 1 | .198** | .207** | 0.011 | .320** |
| Use of instr. support 1 | -0.024 | .222** | .151* | -0.056 | .615** | 0.029 | .293** | 0.142 | .198** | 1 | -0.046 | .192* | 0.09 |
| Substance use 2 | 0.051 | 0.06 | .173* | .762** | 0.113 | .238** | -0.096 | .180* | .207** | -0.046 | 1 | -0.048 | .195* |
| Positive reframing 1 | 0.034 | .168* | 0.087 | 0.038 | 0.072 | -0.039 | .253** | 0.048 | 0.011 | .192* | -0.048 | 1 | 0.028 |
| Self-blame 1 | .156* | -0.102 | .215** | .216** | 0.095 | .475** | -0.05 | .239** | .320** | 0.09 | .195* | 0.028 | 1 |
| Planning 1 | -0.01 | .386** | .187* | -0.113 | .195* | -0.121 | .621** | 0.033 | 0.126 | .376** | -0.092 | .276** | 0.108 |
| Emotional support 2 | -0.019 | .241** | .162* | 0.088 | .612** | 0.053 | .246** | .158* | .222** | .607** | 0.108 | .192* | 0.085 |
| Behavioural disengagement 2 | 0.009 | -0.102 | .329** | .188* | 0.075 | .588** | -0.148 | .435** | .300** | .165* | .162* | -0.013 | .476** |
| Positive reframing 2 | 0.146 | .246** | 0.11 | -0.083 | .150* | -0.088 | .450** | 0.02 | 0.046 | .199** | -0.105 | .542** | -0.017 |
| Humor 1 | 0.013 | 0.106 | .174* | 0.14 | 0.092 | 0.1 | .250** | .160* | .216** | 0.077 | 0.096 | .247** | 0.119 |
| Self distraction 2 | .285** | -0.014 | -0.029 | -0.001 | 0.108 | .210** | 0.114 | 0.015 | 0.113 | 0.054 | -0.046 | .205** | .219** |
| Acceptance 1 | 0.083 | .157* | -0.11 | 0.007 | .238** | -.152* | .299** | -.202** | -0.027 | .173* | -0.049 | .189* | 0.006 |
| Venting 2 | .157* | 0.144 | 0.081 | 0.081 | .411** | .164* | .282** | 0.141 | .321** | .423** | 0.044 | 0.063 | .265** |
| Religion 1 | 0.079 | 0.134 | 0.116 | 0.026 | 0.129 | -0.037 | 0.113 | .181* | 0.104 | 0.125 | -0.056 | .243** | -0.031 |
| Use of instr. support 2 | 0.062 | .316** | .238** | 0.005 | .591** | 0.087 | .317** | .234** | .225** | .707** | 0.048 | .173* | .183* |
| Acceptance 2 | 0.066 | 0.142 | 0.075 | 0.034 | 0.086 | -0.113 | .245** | -0.118 | -0.09 | .195* | -0.112 | .216** | -0.017 |
| Planning 2 | 0.014 | .354** | 0.143 | -0.112 | .237** | -0.057 | .508** | -0.007 | 0.125 | .436** | -0.102 | .217** | 0.1 |
| Self-blame 2 | 0.137 | 0.03 | .265** | 0.122 | 0.138 | .492** | -0.046 | .386** | .288** | .176* | 0.114 | -0.075 | .528** |
| Religion 2 | 0.055 | 0.113 | 0.049 | -0.012 | 0.057 | 0.016 | .191* | 0.121 | 0.126 | 0.122 | -0.007 | .271** | -0.029 |
| Humor 2 | -0.02 | 0.04 | 0.147 | 0.019 | .156* | 0.078 | 0.1 | 0.108 | 0.119 | 0.126 | 0.027 | 0.092 | 0.037 |

|  | Planning 1 | Emotional support 2 | Behavioural diseng. 2 | Positive reframing 2 | Humor 1 | Self distraction 2 | Acceptance 1 | Venting 2 | Religion 1 | Use of info support 2 | Acceptance 2 | Planning 2 | Self-blame 2 | Religion 2 | Humor 2 |
| --- | --- | --- | --- | --- | --- | --- | --- | --- | --- | --- | --- | --- | --- | --- | --- |
| Self distraction 1 | -0.01 | -0.019 | 0.009 | 0.146 | 0.013 | .285** | 0.083 | .157* | 0.079 | 0.062 | 0.066 | 0.014 | 0.137 | 0.055 | -0.02 |
| Active coping 1 | .386** | .241** | -0.102 | .246** | 0.106 | -0.014 | .157* | 0.144 | 0.134 | .316** | 0.142 | .354** | 0.03 | 0.113 | 0.04 |
| Denial 1 | .187* | .162* | .329** | 0.11 | .174* | -0.029 | -0.11 | 0.081 | 0.116 | .238** | 0.075 | 0.143 | .265** | 0.049 | 0.147 |
| Substance use 1 | -0.113 | 0.088 | .188* | -0.083 | 0.14 | -0.001 | 0.007 | 0.081 | 0.026 | 0.005 | 0.034 | -0.112 | 0.122 | -0.012 | 0.019 |
| Emotional support 1 | .195* | .612** | 0.075 | .150* | 0.092 | 0.108 | .238** | .411** | 0.129 | .591** | 0.086 | .237** | 0.138 | 0.057 | .156* |
| Behavioural disengagement 1 | -0.121 | 0.053 | .588** | -0.088 | 0.1 | .210** | -.152* | .164* | -0.037 | 0.087 | -0.113 | -0.057 | .492** | 0.016 | 0.078 |
| Active coping 2 | .621** | .246** | -0.148 | .450** | .250** | 0.114 | .299** | .282** | 0.113 | .317** | .245** | .508** | -0.046 | .191* | 0.1 |
| Denial 2 | 0.033 | .158* | .435** | 0.02 | .160* | 0.015 | -.202** | 0.141 | .181* | .234** | -0.118 | -0.007 | .386** | 0.121 | 0.108 |
| Venting 1 | 0.126 | .222** | .300** | 0.046 | .216** | 0.113 | -0.027 | .321** | 0.104 | .225** | -0.09 | 0.125 | .288** | 0.126 | 0.119 |
| Use of instr. support 1 | .376** | .607** | .165* | .199** | 0.077 | 0.054 | .173* | .423** | 0.125 | .707** | .195* | .436** | .176* | 0.122 | 0.126 |
| Substance use 2 | -0.092 | 0.108 | .162* | -0.105 | 0.096 | -0.046 | -0.049 | 0.044 | -0.056 | 0.048 | -0.112 | -0.102 | 0.114 | -0.007 | 0.027 |
| Positive reframing 1 | .276** | .192* | -0.013 | .542** | .247** | .205** | .189* | 0.063 | .243** | .173* | .216** | .217** | -0.075 | .271** | 0.092 |
| Self-blame 1 | 0.108 | 0.085 | .476** | -0.017 | 0.119 | .219** | 0.006 | .265** | -0.031 | .183* | -0.017 | 0.1 | .528** | -0.029 | 0.037 |
| Planning 1 | 1 | .345** | -0.077 | .303** | .212** | -0.003 | .245** | .342** | 0.072 | .347** | .200** | .653** | -0.01 | 0.076 | 0.084 |
| Emotional support 2 | .345** | 1 | 0.111 | .259** | 0.124 | -0.011 | .237** | .397** | .227** | .620** | 0.144 | .405** | .173* | 0.103 | .187* |
| Behavioural disengagement 2 | -0.077 | 0.111 | 1 | -0.086 | -0.016 | 0.074 | -0.127 | .162* | -0.041 | .180* | -0.053 | -0.002 | .506** | -0.003 | 0.047 |
| Positive reframing 2 | .303** | .259** | -0.086 | 1 | .320** | .318** | .328** | .174* | 0.129 | .262** | .290** | .313** | -0.075 | .220** | .255** |
| Humor 1 | .212** | 0.124 | -0.016 | .320** | 1 | .305** | 0.142 | 0.122 | 0.096 | 0.119 | .214** | .166* | -0.001 | .199** | .673** |
| Self distraction 2 | -0.003 | -0.011 | 0.074 | .318** | .305** | 1 | .182* | .168* | -0.052 | 0.045 | .240** | 0.05 | .207** | -0.001 | .268** |
| Acceptance 1 | .245** | .237** | -0.127 | .328** | 0.142 | .182* | 1 | .317** | 0.119 | .244** | .460** | .332** | -0.135 | 0.063 | 0.145 |
| Venting 2 | .342** | .397** | .162* | .174* | 0.122 | .168* | .317** | 1 | 0.069 | .391** | .186* | .327** | .165* | 0.028 | 0.113 |
| Religion 1 | 0.072 | .227** | -0.041 | 0.129 | 0.096 | -0.052 | 0.119 | 0.069 | 1 | .245** | 0.129 | .221** | 0.04 | .663** | -0.017 |
| Use of instr. support 2 | .347** | .620** | .180* | .262** | 0.119 | 0.045 | .244** | .391** | .245** | 1 | 0.126 | .457** | .300** | 0.11 | 0.135 |
| Acceptance 2 | .200** | 0.144 | -0.053 | .290** | .214** | .240** | .460** | .186* | 0.129 | 0.126 | 1 | .363** | -0.089 | 0.032 | .210** |
| Planning 2 | .653** | .405** | -0.002 | .313** | .166* | 0.05 | .332** | .327** | .221** | .457** | .363** | 1 | 0.041 | 0.133 | 0.104 |
| Self-blame 2 | -0.01 | .173* | .506** | -0.075 | -0.001 | .207** | -0.135 | .165* | 0.04 | .300** | -0.089 | 0.041 | 1 | 0.042 | 0.07 |
| Religion 2 | 0.076 | 0.103 | -0.003 | .220** | .199** | -0.001 | 0.063 | 0.028 | .663** | 0.11 | 0.032 | 0.133 | 0.042 | 1 | 0.023 |
| Humor 2 | 0.084 | .187* | 0.047 | .255** | .673** | .268** | 0.145 | 0.113 | -0.017 | 0.135 | .210** | 0.104 | 0.07 | 0.023 | 1 |

* Correlation is significant at the 0.05 level (2-tailed).

** Correlation is significant at the 0.01 level (2-tailed).
