# Supplementary material for: Are you coping how I'm coping? An exploratory factor analysis of the Brief-COPE among caregivers of children with and without learning disabilities during COVID-19 restrictions in the UK
Source: Int J Dev Disabil. 2024 Jun 4;72(4):717–28. doi: 10.1080/20473869.2024.2359134 (PMC13202675; doi:10.1080/20473869.2024.2359134)
Supplement: Supplemental Material [file YJDD_A_2359134_SM7444.zip › Table vi_Supplementary.docx]

**Table vi**

*Pattern matrix for 7 factor structure*

| **Pattern Matrix^a^** | | | | | | | |
| --- | --- | --- | --- | --- | --- | --- | --- |
|  | Factor | | | | | | |
|  | 1 | 2 | 3 | 4 | 5 | 6 | 7 |
| Eigenvalue | 5.092 | 3.230 | 1.704 | 1.395 | 1.340 | 1.004 | .901 |
| Variance explained (%) | 18.187 | 11.537 | 6.084 | 4.982 | 4.785 | 3.585 | 3.220 |
| Emotional support 1 | **.827** | -.043 | .051 | .089 | .013 | -.129 | .037 |
| Use of instrumental support 1 | **.763** | .056 | .004 | -.130 | .007 | .105 | -.056 |
| Emotional support 2 | **.728** | -.019 | .058 | .059 | .065 | .068 | -.062 |
| Use of instrumental support 2 | **.710** | .140 | -.014 | -.049 | .081 | .146 | -.035 |
| Venting 2 | **.407** | .168 | -.022 | .022 | -.088 | .171 | .241 |
| Behavioural disengagement 1 | -.071 | **.736** | .036 | .018 | -.026 | -.078 | .100 |
| Behavioural disengagement 2 | .064 | **.711** | -.036 | .010 | -.039 | -.073 | .007 |
| Self-blame 2 | .125 | **.694** | -.066 | -.049 | .005 | -.060 | .079 |
| Self-blame 1 | -.009 | **.636** | -.055 | .068 | -.104 | .053 | .268 |
| Denial 2 | .056 | **.549** | .119 | .114 | .176 | .055 | -.277 |
| Denial 1 | -.049 | **.444** | .128 | .109 | .064 | .293 | -.238 |
| Venting 1 | .202 | **.392** | .104 | .064 | .061 | .000 | -.031 |
| Humor 2 | .139 | -.007 | **.825** | -.028 | -.093 | -.125 | -.028 |
| Humor 1 | -.071 | .020 | **.809** | .094 | .068 | .076 | .014 |
| Substance use 1 | -.031 | -.041 | .008 | **.938** | -.013 | -.022 | .101 |
| Substance use 2 | .028 | .023 | .018 | **.812** | -.068 | -.046 | -.023 |
| Religion 1 | .110 | -.026 | -.094 | -.022 | **.839** | -.077 | .000 |
| Religion 2 | -.033 | .015 | .009 | -.039 | **.786** | -.039 | .010 |
| Positive reframing 1 | -.048 | -.045 | .142 | -.007 | .286 | .241 | .193 |
| Planning 1 | .049 | .046 | -.026 | -.080 | -.113 | **.856** | -.063 |
| Active coping 2 | -.022 | -.035 | .053 | -.029 | .031 | **.737** | .043 |
| Planning 2 | .203 | .029 | -.053 | -.114 | .017 | **.626** | .079 |
| Active coping 1 | .119 | -.078 | -.036 | .125 | .073 | **.447** | -.041 |
| Positive reframing 2 | .003 | -.082 | .265 | -.080 | .181 | .320 | .287 |
| Self distraction 2 | -.050 | .192 | .291 | -.096 | -.039 | -.083 | **.566** |
| Acceptance 1 | .218 | -.276 | .042 | .036 | .001 | .168 | **.452** |
| Acceptance 2 | .054 | -.162 | .148 | -.008 | .012 | .214 | .349 |
| Self distraction 1 | -.002 | .128 | -.062 | .072 | .077 | -.009 | .329 |
