# Supplementary material for: Are you coping how I'm coping? An exploratory factor analysis of the Brief-COPE among caregivers of children with and without learning disabilities during COVID-19 restrictions in the UK
Source: Int J Dev Disabil. 2024 Jun 4;72(4):717–28. doi: 10.1080/20473869.2024.2359134 (PMC13202675; doi:10.1080/20473869.2024.2359134)
Supplement: Supplemental Material [file YJDD_A_2359134_SM7444.zip › Table v_Supplementary.docx]

**Table v**

*Pattern matrix for 6 factor structure*

|  | | | | | | |
| --- | --- | --- | --- | --- | --- | --- |
|  | Factor | | | | | |
|  | 1 | 2 | 3 | 4 | 5 | 6 |
| Eigenvalue | 5.065 | 3.211 | 1.591 | 1.360 | 1.310 | .980 |
| Variance explained (%) | 18.09 | 11.47 | 5.68 | 4.86 | 4.68 | 3.50 |
| Emotional support 1 | **.820** | -.036 | .018 | .108 | .028 | .118 |
| Use of instrumental support 1 | **.752** | .073 | -.066 | -.117 | .030 | -.118 |
| Use of instrumental support 2 | **.715** | .154 | -.058 | -.042 | .095 | -.151 |
| Emotional support 2 | **.708** | .004 | -.031 | .078 | .089 | -.090 |
| Venting 2 | **.450** | .145 | .151 | .002 | -.110 | -.132 |
| Behavioural disengagement 1 | -.063 | **.727** | .119 | .002 | -.047 | .086 |
| Behavioural disengagement 2 | .067 | **.715** | -.019 | -.009 | -.050 | .071 |
| Self-blame 2 | .144 | **.684** | .008 | -.073 | -.015 | .070 |
| Self-blame 1 | .048 | **.589** | .160 | .031 | -.142 | -.007 |
| Denial 2 | .012 | **.585** | -.085 | .129 | .200 | -.099 |
| Denial 1 | -.080 | **.476** | -.038 | .121 | .087 | -.322 |
| Venting 1 | .187 | **.404** | .060 | .074 | .067 | -.017 |
| Self distraction 2 | -.017 | .135 | **.678** | -.114 | -.084 | .145 |
| Humor 1 | -.141 | .060 | **.594** | .139 | .110 | -.142 |
| Humor 2 | .011 | .045 | **.517** | .046 | -.014 | -.004 |
| Positive reframing 2 | .018 | -.104 | **.489** | -.083 | .169 | -.275 |
| Acceptance 2 | .091 | -.191 | **.396** | -.026 | -.014 | -.164 |
| Acceptance 1 | .275 | -.311 | **.360** | .008 | -.037 | -.102 |
| Positive reframing 1 | -.028 | -.059 | .313 | -.018 | .272 | -.199 |
| Self distraction 1 | .057 | .085 | .204 | .039 | .030 | .067 |
| Substance use 1 | .006 | -.026 | .059 | **.863** | -.034 | .045 |
| Substance use 2 | .039 | .022 | -.032 | **.857** | -.080 | .039 |
| Religion 1 | .127 | -.017 | -.044 | -.038 | **.809** | .087 |
| Religion 2 | -.028 | .023 | .056 | -.047 | **.786** | .053 |
| Planning 1 | .081 | .058 | -.026 | -.090 | -.113 | **-.843** |
| Active coping 2 | .012 | -.034 | .132 | -.041 | .026 | **-.701** |
| Planning 2 | .250 | .024 | .047 | -.133 | .005 | **-.588** |
| Active coping 1 | .144 | -.070 | -.036 | .122 | .072 | **-.431** |
